# Estimating Foodborne Gastroenteritis, Australia

**DOI:** 10.3201/eid1108.041367

**Published:** 2005-08

**Authors:** Gillian Hall, Martyn D. Kirk, Niels Becker, Joy E. Gregory, Leanne Unicomb, Geoffrey Millard, Russell Stafford, Karin Lalor, the OzFoodNet Working Group

**Affiliations:** * Australian National University, Canberra, Australian Capital Territory (ACT), Australia;; † OzFoodNet, Canberra, ACT, Australia;; ‡ Department of Human Services, Melbourne, Victoria, Australia;; § Hunter Population Health Unit, Wallsend, New South Wales, Australia;; ¶ ACT Analytical Laboratory, Weston Creek, ACT, Australia;; # Queensland Health, Archerfield, Queensland, Australia

**Keywords:** gastroenteritis, foodborne, burden, uncertainty

## Abstract

We estimated for Australia the number of cases, hospitalizations, and deaths due to foodborne gastroenteritis in a typical year, circa 2000. The total amount of infectious gastroenteritis was measured by using a national telephone survey. The foodborne proportion was estimated from Australian data on each of 16 pathogens. To account for uncertainty, we used simulation techniques to calculate 95% credibility intervals (CrI). The estimate of incidence of gastroenteritis in Australia is 17.2 million (95% confidence interval 14.5–19.9 million) cases per year. We estimate that 32% (95% CrI 24%–40%) are foodborne, which equals 0.3 (95% CrI 0.2–0.4) episodes per person, or 5.4 million (95% CrI 4.0–6.9 million) cases annually in Australia. Norovirus, enteropathogenic *Escherichia coli*, *Campylobacter* spp., and *Salmonella* spp. cause the most illnesses. In addition, foodborne gastroenteritis causes ≈15,000 (95% CrI 11,000–18,000) hospitalizations and 80 (95% CrI 40–120) deaths annually. This study highlights global public health concerns about foodborne diseases and the need for standardized methods, including assessment of uncertainty, for international comparison.

## Abstract 2

An estimated 4.0–6.9 million episodes of foodborne gastroenteritis occur in Australia each year.

The pattern of foodborne disease has changed substantially in industrialized countries in recent decades. Outbreaks are more likely to be far reaching, and some are even global in scale because of widespread food distribution and changes in methods of food preparation (1). Further changes in the incidence of foodborne disease and the pattern of food-related illness can be anticipated from global warming (2). As a result of changed conditions in food production and better laboratory detection techniques, new foodborne pathogens continue to be identified (3). In particular, we are now faced with the emergence of antimicrobial drug–resistant bacteria and a number of viruses not previously recognized (4,5).

Foodborne disease is a public health concern in all parts of the world. In the United States, foodborne disease causes an estimated 76 million illnesses, 325,000 hospitalizations, and 5,000 deaths each year (6). In the United Kingdom, an estimated 2.37 million cases of foodborne gastroenteritis occurred in 1995 (7). Previous estimates of foodborne gastroenteritis in Australia have ranged from 1–2 million (8) to 4 million episodes per year (9). The effect of such large numbers of persons with gastrointestinal illness is considerable. A recent national survey of gastroenteritis in Australia found that one third of working adults miss ≥1 days of work when they have gastroenteritis, and another third of cases result in a caregiver missing work (10). In Australia in 2003, 99 reported outbreaks of foodborne disease affected 1,686 people and caused 6 deaths (11). Any evidence of food contamination can also have a major effect on food industry and trade. The food industry in Australia generates >$29 billion in food production, with >20% of products exported, and $57 billion in food processing (12).

## Transmission of Pathogens Causing Gastroenteritis

Infectious gastroenteritis is caused by many pathogens, each with unique characteristics requiring different laboratory tests for identification. Transmission of pathogens to humans may occur from contaminated foods or water, or from infected persons, environments, or animals. Some pathogens that cause gastroenteritis, such as *Bacillus cereus*, are always thought to be the result of contaminated food, whereas others, such as rotavirus, are largely transmitted by nonfoodborne routes. Most enteric pathogens have multiple modes of transmission (13).

Many clinical cases of gastroenteritis are assessed as "presumed infectious" and do not have a pathogen isolated, even when a stool sample is tested (14). These include cases in which a known pathogen is present but is not identified and cases in which the pathogen is totally unknown (15). Numerous pathogens that were unknown only a few decades ago are now considered commonplace, including *Campylobacter* spp., Shiga toxin–producing *Escherichia coli*, and norovirus (16). More unknown pathogens are likely to be major causes of illness, some of which will become apparent with time and investigation.

## Estimating the Level of Foodborne Gastroenteritis

As part of the activities of OzFoodNet, the foodborne disease surveillance network operating in Australia since 2000, we undertook a study to estimate the amount of foodborne gastroenteritis in contemporary Australia. In the absence of an internationally agreed-upon methodology, the approach used in the United States provided the best method for obtaining internationally comparable estimates (6). Two components are required to estimate the extent of foodborne gastroenteritis by this method: 1) the total amount of gastroenteritis in the country and 2) the proportion of gastroenteritis that is foodborne. The product of these 2 estimates gives the total number of cases of foodborne gastroenteritis.

Uncertainty is inherent in data used in such calculations. Variability may be quantified by statistical concepts like standard error and confidence intervals, but other components of uncertainty are due to paucity of data. Since some information is always available, however, we can quantify each component of uncertainty by a plausible probability distribution using all relevant information available. We can then use simulations of these distributions to generate an interval that contains the credible estimates of the number of foodborne cases of gastroenteritis. These interval estimates are akin to credibility intervals used in Bayesian inferences.

Our objective for this study was to use Australian data to calculate the number of cases, hospitalizations, and deaths due to foodborne gastroenteritis in Australia in a typical year, around the year 2000, accounting for uncertainty in the estimate.

## Methods

The calculations to estimate incidence, hospitalizations, and deaths are described below and in the Figure. Details of the data sources and the simulation technique used to account for uncertainty are shown in the Appendix.

We considered the definition of foodborne to include any infectious gastroenteritis caused by eating food, including food contaminated just before eating. The proportion of infectious gastroenteritis cases that are due to foodborne transmission in the community and their proportion among hospitalizations and deaths, were assessed by using data from multiple Australian sources about individual known pathogens.

Gastroenteritis caused by known pathogens was studied to estimate the proportion of all infectious gastroenteritis that is foodborne. Of 25 pathogens with the potential for foodborne transmission, 16 (those listed in Table 1) were considered relevant. Pathogens not considered relevant were either not foodborne (*Clostridium difficile*), did not cause gastroenteritis (*Brucella* spp., *Listeria* spp., *Toxoplasma gondii*, hepatitis A virus), or were only acquired overseas (*Salmonella* Typhi, *Vibrio cholerae*, *Cyclospora cayetanensis*, *Trichinella spiralis*).

**Table 1:** GE due to known pathogens in Australia in a typical year circa 2000*

Pathogen	Median no. GE cases (95% CrI)	Median proportion foodborne (95% CrI)	Median no. foodborne (95% CrI)
Bacteria			
*Aeromonas* spp.	39,400 (31,700–47,200)	0.25 (0.12–0.38)	9,800 (4,100–15,400)
*Bacillus cereus*	6,900 (0–16,000)	1 (1.00–1.00)	6,900 (0–15,800)
*Campylobacter* spp.	277,000 (89,800–463,000)	0.75 (0.67–0.83)	208,000 (67,000–350,000)
*Clostridium perfringens*	43,000 (440–86,000)	1 (1.00–1.00)	43,000 (400–86,000)
STEC	3,000 (0–6,500)	0.65 (0.48–0.82)	1,900 (0–4,200)
non-STEC *E. coli*	1,152,000 (797,000–1,507,000)	0.5 (0.32–0.68)	563,000 (295,000–831,000)
*Salmonella* spp.	92,000 (26,000–158,000)	0.87 (0.81–0.93)	81,000 (23,000–138,000)
*Shigella* spp.	3,200 (0–6,900)	0.1 (0.04–0.16)	300 (0–700)
*Staphylococcus aureus*	14,100 (0–29,800)	1 (1.00–1.00)	14,200 (0–29,800)
*Vibrio parahaemolyticus*	1,080 (0–2,600)	0.71 (0.54–0.88)	740 (0–1,850)
*Yersinia* spp.	2,200 (0–4,500)	0.75 0.63–0.87)	1,620 (0–3,400)
Total bacteria	1,639,000 (1,175,000–2,103,000)	0.58 (0.44–0.72)	950,000 (590,000–1,310,000)
Viruses			
Norovirus	1,832,000 (1,361,000–2,302,000)	0.25 (0.12–0.38)	446,000 (193,000–700,000)
Rotavirus	241,000 (98,000–384,000)	0.02 (0.01–0.03)	4,700 (700–8,600)
Astrovirus/adenovirus	190,000 (63,000–316,000)	0.1 (0.02–0.18)	17,500 (0–36,800)
Total viruses	2,280,000 (1,740,000–2,820,000)	0.21 (0.11–0.31)	470,000 (210,000–730,000)
Parasites			
*Cryptosporidium parvum*	271,000 (255,000–287,000)	0.1 (0.02–0.18)	25,000 (0–54,000)
*Giardia lamblia*	430,000 (232,000–628,000)	0.05 (0.01–0.09)	20,400 (0–41,100)
Total parasites	704,000 (442,000–966,000)	0.14 (0.04–0.24)	66,000 (18,000–114,000)
Total	4,640,000 (3,750,000–5,510,000)	0.32 (0.24–0.40)	1,480,000 (1,030,000–1,920,000)
*GE, gastroenteritis; CrI, credibility interval; STEC, Shiga toxin–producing *Escherichia coli*.			

For each of the 16 pathogens, yearly estimates were made of the total number of cases of gastroenteritis in the community; this number was based on data collected in the National Notifiable Diseases Surveillance System, published results from the Water Quality Study conducted in Melbourne in 1998 (17,18), laboratory data, or outbreak data. Necessary adjustments were made for underreporting in the Australian surveillance system, incomplete population coverage, and the proportion of infections acquired overseas (Appendix). We also estimated, for each of the pathogens, the total number of hospitalizations and deaths based on data from the National Hospital Morbidity Database (NHMD) as well as the proportion of cases due to foodborne transmission, which was estimated from outbreak data, the literature, and because Australian data were lacking, a Delphi process involving 10 foodborne disease experts in Australia. The number of foodborne episodes for each pathogen was then obtained by multiplying the estimate of the foodborne proportion by the estimate of the total number of cases, hospitalizations, and deaths. The overall proportion of infectious gastroenteritis in the community due to foodborne transmission was then estimated by dividing the sum of the foodborne cases due to the 16 known pathogens by the sum of all cases due to the 16 known pathogens (Figure). The equivalent calculation was also done for hospital admissions due to known pathogens to give the proportion of hospitalizations for infectious gastroenteritis due to foodborne transmission.

We assumed that the proportion of gastroenteritis that is foodborne is the same among cases caused by known pathogens as among those caused by unknown pathogens. Adjustments were made for the proportion estimated as acquired overseas for certain pathogens (Appendix).

### Incidence of Foodborne Gastroenteritis

The total amount of infectious gastroenteritis in Australia in 1 year was estimated from the National Gastroenteritis Survey 2001–2002. This computer-assisted telephone survey ran during 12 months from September 2001 to August 2002 in all states and territories of Australia. Ethics clearance was obtained from the Australian Department of Health and Ageing Ethics Committee and from other state health departments and university committees. Random digit dialing was used to select households, and then the person with the most recent birthday was selected as the respondent. The response rate was 67%, and the final sample was 6,087 persons. Data were collected on symptoms of gastroenteritis in the previous 4 weeks. The case definition excluded persons who identified a noninfectious cause for their symptoms, and an adjustment was made for persons with gastrointestinal symptoms secondary to a respiratory infection (19). The case definition was ≥3 loose stools or ≥2 episodes of vomiting or, if respiratory symptoms were present, ≥4 loose stools or ≥3 episodes of vomiting in a 24-hour period in the previous 4 weeks.

The final estimate of the total number of cases of foodborne gastroenteritis in the community is the product of the proportion foodborne and the total number of cases of infectious gastroenteritis. The incidence per person per year was estimated by using population data from the Australian Bureau of Statistics (20).

### Hospitalizations for Foodborne Gastroenteritis

The total number of hospitalizations for gastroenteritis was estimated from the National Hospital Morbidity Database (21), which records all admissions to hospital in Australia. Data from 1993/1994 to 1998/1999 were examined for International Classification of Diseases, Ninth Revision, Clinical Modification (ICD-9-CM) codes for gastroenteritis as either a principal diagnosis of as any of a further 9 additional diagnoses. Additional diagnoses refer to cases in which gastroenteritis was a contributing factor to, but not the only reason for, admission. These data were used to estimate the average number of diagnoses per year for each of 14 of the known pathogens. No ICD code for *Aeromonas* infection was in the hospital separation dataset, and hospitalizations due to *E. coli* infections did not distinguish between enteropathogenic and Shiga toxin–producing types, leading to a study of 14 rather than 16 known pathogens in hospital data. Diagnoses coded "gastroenteritis, presumed infectious" were also examined. Adjustments were not made for underreporting of individual pathogens, since any missed diagnoses for specific pathogens should be included in this unknown category.

The final estimate of the total number of hospitalizations due to foodborne gastroenteritis was the product of the proportion of hospitalizations among known pathogens that is foodborne and the total number of hospitalizations for infectious gastroenteritis (including cases of unknown but presumed infectious causes).

### Deaths Due to Foodborne Gastroenteritis

Deaths in the hospital were determined for gastroenteritis as either a principal diagnosis of as any of a further 9 additional diagnoses (data from 1993/1994 to 1998/1999). The final estimate of the total number of deaths due to foodborne gastroenteritis was the product of the proportion foodborne and the total number of deaths in the hospital due to infectious gastroenteritis.

### Accounting for Uncertainty in Data

Where suitable data were available, 95% confidence intervals (CI) were calculated. Otherwise, uncertainty in the data was accounted for by using simulation techniques. Plausible probability distributions were generated by using all available information, and the interval between 2.5th and 97.5th percentiles gave the 95% credible interval (95% CrI). The median was taken as the point estimate. This technique is explained in the Appendix. Calculations were carried out with the Statistical Package for the Social Sciences, version 11.50 (SPSS Inc., Chicago, IL, USA).

## Results

### Incidence

The case definition applied to 450 (7%) of 6,087 respondents to the National Gastroenteritis Survey. When weighted to the Australian population by age and sex, this number extrapolated to 17.2 million (95% CI 14.5–19.9 million) cases of gastroenteritis in Australia in 1 year, or 0.92 (95% CI 0.77–1.06) cases per person per year (13). This number includes all causes of infectious gastroenteritis.

Among the 16 known pathogens were an estimated 4.6 million (95% CrI 3.8–5.5 million) cases of gastroenteritis due to all modes of transmission. Of these, an estimated 1.6 million (95% CrI 1.2–2.1 million ) were due to bacterial infections, 2.3 million (95% CrI 1.7–2.8 million) were due to viral infections, and 0.70 million (95% CrI 0.44–0.97 million) were due to parasites (Table 1).

Among known pathogens, 1.5 million (95% CrI 1.0–1.9 million) cases were acquired through food. Enteropathogenic *E. coli*, noroviruses, *Campylobacter* spp., and *Salmonella* spp. accounted for 88% of all foodborne disease in this group of pathogens (Table 1). The proportion of gastroenteritis due to foodborne transmission was estimated at 32% (95% CrI 24–40%). The product of the total number of cases of gastroenteritis (17.2 million; 95% CrI 14.5–19.9 million) multiplied by the proportion that was foodborne (0.32, 95% CrI 0.24–0.40) produced an estimate of 5.4 million cases of foodborne gastroenteritis in 1 year in Australia (circa 2002), with a 95% CrI of 4.0–6.9 million cases.

### Hospitalizations

Among hospitalizations for gastroenteritis due to the 14 known pathogens were 10,070 (95% CrI 8,630–11,470) diagnoses of gastroenteritis; an estimated 3,640 (95% CrI 2,600–4,670) of these cases were due to eating contaminated food. The overall proportion of hospitalizations estimated to be from foodborne gastroenteritis was 0.36 (95% CrI 0.30–0.41) (Table 2).

**Table 2:** Estimated hospital diagnoses of gastroenteritis due to foodborne pathogens in Australia, circa 2000*

Agent	ICD-9-CM code	No. hospital diagnoses per year (95% CrI)†	% foodborne (95% CrI)	No. hospital diagnoses from foodborne transmission per year (95% CrI)‡
Bacteria		4,960 (3,360–6,310)	70 (65–75)	3,480 (2,440–4,500)
*Aeromonas* spp.	NA	NA	25 (12–38)	–
*Bacillus cereus*	008.59	29 (0–66)	100	29 (0–66)
*Campylobacter* spp.	008.43	3,140 (1,754–4,546)	75 (67–83)	2,260 (1,250–3,300)
*Clostridium perfringens*	005.2	1 (0–3)	100	1 (0–3)
*Escherichia coli*	008.00–04	102 (53–154)	50 (32–68)	50 (23–86)
*Salmonella* spp. (nontyphoidal)	003	1,330 (1,130–1,530)	87 (81–93)	1,060 (900–1,240)
*Shigella* spp.	004	320 (270–370)	10 (4–16)	19 (8–31)
*Staphylococcus aureus*	005.0	21 (17–25)	100	21 (17–25)
*Vibrio parahaemolyticus*	005.4	4 (2–6)	71 (54–88)	3 (1–5)
*Yersinia enterocolitica*	008.44	34 (24–44)	75 (63–87)	25 (17–35)
Viruses		3,940 (3,740–4,140)	2 (1–3)	100 (60–140)
Astrovirus/adenovirus	008.62/008.66	190 (130–250)	10 (2–18)	19 (4–37)
Norovirus	008.63	17 (2–32)	25 (12–38)	4 (0–9)
Rotavirus	008.61	3,740 (3,540–3,920)	2 (1–3)	70 (40–110)
Parasites		1,160 (950–1,390)	6 (2–9)	64 (19–116)
*Cryptosporidium* spp.	007.4	200 (0–400)	10 (2–18)	14 (0–49)
*Giardia lamblia*	007.1	1,000 (900–1,100)	5 (1–9)	49 (7–90)
Total known§		10,070 (8,630–11,470)		3,640 (2,600–4,670)
Miscellaneous and unknown agents		30,800 (22,700–38,400)	36 (30–41)¶	11,000 (8,000–14,000)
Miscellaneous agents (not listed above)		2,800 (2,400–3,200)		1,000 (800–1,200)
Unknown#		28,000 (20,000–35,700)		10,000 (6,800–13,200)
Total known and unknown		41,000 (33,000–49,000)	36 (30–41)	14,700 (11,400–18,000)

*ICD-9-CM, International Classification of Diseases, Ninth Revision, Clinical Modification; CrI, credibility interval; NA, not applicable. †Includes principal and 9 additional diagnoses. Simulated distribution based on raw yearly National Hospital Mortality Database data 1993/1994–1998/1999. ‡Adjusted for 1) proportion foodborne and 2) proportion overseas acquired. §36% (95% CrI 30%–41%) known pathogens foodborne (not overseas acquired). ¶Apply % foodborne in known pathogens. #Includes codes 0051–3 and 8–9, 00849, 0085, 0088, 009, 00841–2, 00846–9,and 00869.

The total number of hospital diagnoses for gastroenteritis was estimated as 41,000 (95% CrI 33,000–49,000). The number due to foodborne transmission was 14,700 (95% CrI 11,400–18,000) (Table 3).

**Table 3:** Infectious and foodborne gastroenteritis in Australia in a typical year, circa 2000*

Measure	All causes estimate (95% CrI)	Foodborne transmission estimate (95% CrI)
No. cases per year (×10^6^)	17.2 (14.5–19.9)	5.4 (4.0–6.9)
Known pathogen	4.6 (3.7–5.5)	1.5 (1.0–1.9)
Cases per person per year	0.92 (0.77–1.06)	0.29 (0.23–0.35)
No. hospital diagnoses per year (×10^3^)	40.9 (32.7–48.6)	14.7 (11.4–17.7)
Known pathogen	10.1 (8.6–11.5)	3.6 (2.6–4.7)
Hospital diagnoses per 10,000 persons per year	22 (17–26)	8 (6–9)
Deaths per year	217 (120–320)	76 (40–120)
Deaths per 10,000 persons per year	0.12 (0.06–0.17)	0.04 (0.02–0.06)

*CrI, credibility interval.

### Deaths

The NHMD 1993/1994 to 1998/1999 showed 1,302 deaths (157–311 per year) in patients with a code for a principal or additional diagnosis of infectious gastroenteritis in the 6 years. The average was 217 (standard deviation 51) per year. Of these 1,302 deaths, 287 occurred in patients with a principal diagnosis of infectious gastroenteritis. Application of the proportion of hospital diagnoses due to foodborne gastroenteritis (36%, 95% CrI 30%–41%) to the number of deaths in which the diagnosis included infectious gastroenteritis (217, 95% CrI 120–320) provided an estimate of 76 (95% CrI 41–120) deaths due to foodborne gastroenteritis each year (Table 3).

## Discussion

The estimates from this study demonstrate the considerable prevalence of foodborne disease in contemporary Australia and justify the attention given to foodborne disease surveillance and food safety. The uncertainty estimates indicate that even the lower boundary of the credible interval is still high, with at least 4 million cases of foodborne gastroenteritis, and possibly as many as 7 million per year. This means that on average, every Australian can expect to experience an episode of foodborne illness about every 3 to 4 years. Hospitalizations are uncommon at 8 per 10,000 people each year, and ≈4 deaths per million persons occur per year.

Similar studies have been done in United States (6) and the United Kingdom (7). The Australian estimate of incidence is remarkably similar to that reported for the United States, but higher than in the United Kingdom. In the United States, 36% of all gastroenteritis was estimated to be due to foodborne transmission, and incidence was estimated at 0.28 cases per person per year. In the United Kingdom, 26% of gastroenteritis was estimated to be due to foodborne transmission, and incidence was estimated at 0.04 cases per person per year in 1995. The importance of using a standardized method when comparing results of the amount of foodborne gastroenteritis across countries or times cannot be overemphasized. Evidence suggests that a prospective cohort study design may produce a lower incidence of community gastroenteritis than a cross-sectional design. The UK study included a quality control substudy to compare the incidence based on a retrospective recall method with incidence from a prospective diary method; the estimates of incidence were 0.6 and 0.2 cases per person per year, respectively (14). Prospective studies that require participants to supply a stool sample every time they report gastroenteritis might tend to cause an underestimate because of unwillingness to provide a sample; on the other hand, in a retrospective recall method, respondents might "telescope" events into a shorter time frame. A prospective study done in the Netherlands (22) also found a lower incidence than that seen in the United Kingdom. Other variations in methods also exist across countries, such as differences in surveillance systems and the quality of outbreak data available to estimate the proportion of cases that are foodborne. Not only may the study design influence the final estimate, but also the definition of gastroenteritis. Even a seemingly small difference in the definition of gastroenteritis can lead to a considerable difference in the final estimates (23).

The definition of community gastroenteritis used in this Australian study refers to moderate-to-severe illness, with at ≥3 loose stools or ≥2 episodes of vomiting in a single day. To improve the specificity of our definition for enteric illness, we excluded patients with concomitant respiratory symptoms unless they had more severe symptoms of diarrhea or vomiting. Previous studies have found similarly high rates of respiratory symptoms amongst cases of gastroenteritis (24). A definition inclusive of milder illness would lead to a higher estimate of foodborne gastroenteritis, and a definition that included only more severe illness would lead to a lower estimate.

We also took account of those with concurrent respiratory symptoms in our definition of community gastroenteritis, although most studies estimating the amount of gastroenteritis have not considered this. The United States study (6) adjusted for those with respiratory illness by excluding a proportion of case-patients who were thought likely to have symptoms secondary to respiratory infections rather than a primary enteric infection. The UK definition of gastroenteritis was different from the Australian definition in several ways. While differing arguments can be raised about the best definition of gastroenteritis, the main concern is to have a consistent, reasonable definition for comparative purposes.

Of the 5.4 million (95% CrI 4.0–6.9 million) cases of foodborne gastroenteritis, 28% were attributed to known pathogens. This finding compares with 18% in the United States (6) and 41% in the United Kingdom (7). The Australian data used to estimate the pathogen-specific numbers of community cases of gastroenteritis were variable in quality. *Salmonella* notifications have been relatively stable over the last 5 years, and characteristics of this illness are fairly well understood. In comparison, reports of illness due to *Campylobacter* have increased steadily during the same time (25). This finding could be due to reporting artifacts or increasing infection rates in the community. The diagnostic laboratory tests have not changed appreciably during this time.

The pathogen-specific estimates in this study that most influenced the final estimate of the proportion of gastroenteritis that is foodborne were those for norovirus and enteropathogenic *E. coli*, as these accounted for the largest numbers. The estimates for both were determined from a high-quality longitudinal study (17). Nevertheless, the sample was limited to a specific subpopulation and geographic location. The high proportion of *E. coli* is similar to findings in the United Kingdom, although we estimated that 50% of cases caused by this pathogen were foodborne compared to 8% of cases in the UK assessment (7) and 30% in the United States (6). In recent years, the capacity of laboratories to identify noroviruses with polymerase chain reaction tests has improved considerably, and this virus is likely to become increasingly recognized (26).

Factors were used to adjust for underreporting when using data from outbreaks and surveillance. Further studies are needed to give more robust estimates of the level of underreporting compared with the true level in the community. The estimates of the proportion of illness due to foodborne transmission for specific pathogens relied largely on outbreak data and opinions of foodborne diseases experts. Outbreak data can be very sensitive to the outcomes from larger events, which could bias the estimate of the proportion foodborne in either direction (27). Foodborne disease experts' experience was based on pathogen characteristics in the laboratory, results of outbreak investigations, and knowledge from case-control studies of sporadic infections. For pathogens estimated to have a large number of cases, such as norovirus, the estimate of the proportion thought to be foodborne can influence the final estimate. Both the UK and Australian estimates were based on outbreak data, but only 11% of Norwalk-like virus (caliciviruses) gastroenteritis was ascribed to foodborne transmission in the UK study, compared with 40% in the United States, and 25% in Australia (6,7). These individual estimates had some influence on the final estimates of the proportion of all gastroenteritis that is foodborne.

Hospital data in Australia are fairly complete, and only a few hospitals, mostly private, have not contributed records of all admissions to the national database in the last decade (21). Coding of admissions varies over time and place, but a patient with gastroenteritis is likely to be coded for this condition in the first 10 diagnoses (28). Approximately two thirds of diagnoses were coded as the main reason for admission. Additional diagnoses may represent cases with complications or comorbidity that took precedence in the order of coding or cases acquired in the hospital. Some deaths due to gastroenteritis may have occurred in nursing homes, which were not included. Among the known pathogens, bacterial infections accounted for >90% of hospital admissions in Australia, which is similar to the proportion in the United Kingdom (7) but higher than the 60% estimated in the United States (6). Campylobacteriosis followed by salmonellosis accounted for most admissions due to bacterial infections in Australia and the United Kingdom; in the United States this order was reversed. These illnesses are important when considering the severe end of the spectrum of foodborne gastroenteritis.

We used the best available Australian data to conduct this study, but as experienced by others conducting research overseas, the quality of the data inevitably varied. Since the quality of the data cannot be easily improved, we chose to provide estimates that reflect the true state of uncertainty of the data by using a simulation technique that can be easily applied. Taking account of uncertainty informs the data users, including policy makers, that a very precise estimate is not possible. An appreciation of the degree of confidence that can be placed in an estimate is an important part of the responsible presentation of results that may have considerable effects at a policy level. With these stipulations, we are confident that the level of foodborne gastroenteritis is high in Australia. In the future, improvements in data completeness and quality would enhance the robustness of the calculations, but estimates of uncertainty are likely to remain an important component of the results.

## Appendix

### Data and Adjustment Factors

Raw data sources and adjustments required are shown in Table A1.

#### Adjustment for Underreporting

In the calculations for determining the underreporting factor from the community, the sensitivity of laboratory tests was estimated to be ≈90% (on the basis of estimates for *Salmonella* and *Shigella* testing in Australia), and laboratory reporting was estimated at 100%. The underreporting factors were based on information from various sources as outlined in Table A2.

#### Adjustment for Overseas-acquired Cases

Notifiable data from the states of Victoria and South Australia were used to estimate the proportion of cases that were acquired overseas for different pathogens as shown in Table A1. The relevant proportion of cases was subtracted from the estimates.

#### Adjustment for Underreporting of Outbreaks Compared with Surveillance

The outbreak factor describes the relationship between the number of cases identified in outbreaks, and the number of cases that would have been identified by surveillance had the disease been a notifiable illness. Since the pathogens of interest are not actually reported to surveillance, the outbreak factor was based on *Salmonella* data that were reported to both surveillance and a database of outbreaks in Victoria from 1998 to 2002. The plausible distribution of the outbreak factor was deduced from a comparison of the number of *Salmonella* notifications and the number of *Salmonella* cases identified in outbreaks.

On average, we found 14 times as many notifications as cases identified in outbreaks in Victoria, with variability each year. The outbreak factor was simulated as a normal distribution with a mean of 14 and a standard deviation of 4. The illness severity for the 3 pathogens, based on outbreak data, was moderate, so the underreporting factor for moderate illness was used.

### Simulation Technique to Account for Uncertainty

Wherever uncertainty existed for a factor used in the calculations, a simulated distribution of plausible values was used to model the uncertainty in that factor, rather than a point estimate. In the absence of definitive, statistically sound data, decisions about the plausible distribution of values are based on a reasonable interpretation of real data. In other words, the parameters of the plausible distribution are not necessarily based on a statistically derived value but on judgments guided by the best available data. The different distributions are used to simulate 1,000 plausible values, and the most likely values have the greatest frequency. The median was used as the central estimate, and the credible interval was taken as the 2.5–97.5 percentile of the simulated distributions. The width of the credible interval of the final estimate is therefore determined by the precision with which each of the component probabilities is estimated.

**Figure A1:**
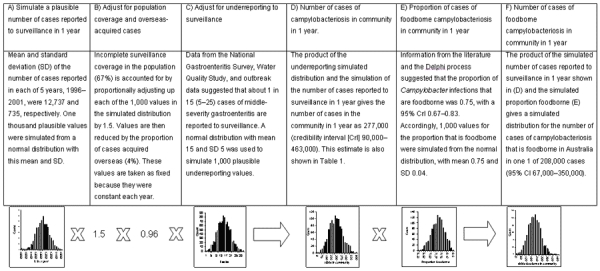
Estimate of the number of foodborne cases of *Campylobacter* infection, with simulation to account for uncertainty.

An example of the simulation technique is shown in the Figure A1. It shows the estimation of the number of community cases of campylobacteriosis by using Notifiable Diseases Surveillance data, leading to the estimate of the number of foodborne cases of *Campylobacter* infection for Australia.

## Figures and Tables

**Figure:**
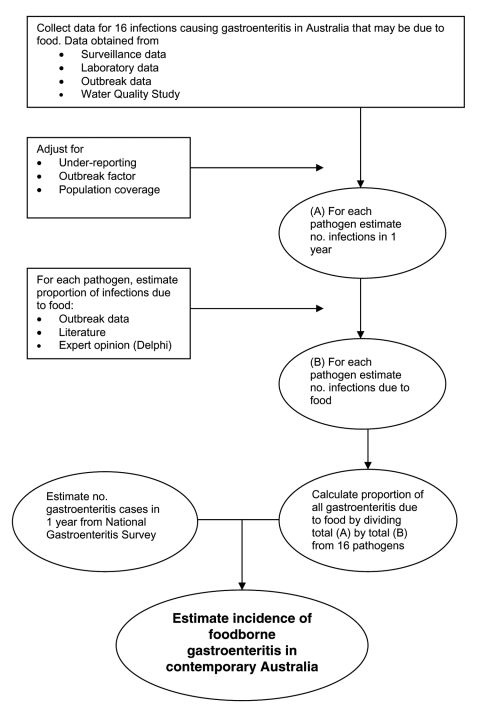
Scheme of data sources and calculations used to estimate the number of cases of foodborne gastroenteritis in the community in 1 year in Australia around the year 2000.

## Appendix

**Table A1:** Sources of raw data and adjustments for each microorganism

Source/pathogen	Raw data
Water Quality Study 1998–1989, Melbourne	No. positive/no. stools tested
*E. coli*, other (pathogenic, non-STEC)†	53/791
Calicivirus†	75/703
Rotavirus†	11/791
Astrovirus/adenovirus†	9/791
* Cryptosporidium parvum†*	13/791
* Giardia lamblia†*	20/791
Queensland and South Australian laboratory data, various years 1995–2001	No. positive/no. stools tested
*Aeromonas* spp. †	248/107,600
* Vibrio parahaemolyticus†*	2/30,880
Victorian outbreaks, 4-year data, 1998–2001	Mean no. cases/y (range)
*Bacillus cereus*‡§	12 (0–37)
*Clostridium perfringens*‡§	60 (28–73)
*Staphylococcus aureus*‡§	15 (0–40)
National Notifiable Diseases Surveillance System, 5-year data, 1996–2000	Mean no. cases/y (range)
*Campylobacter* spp. (AOA 4%)‡§	Not including NSW, 12,756 (11,829–13,528)
*Salmonella* spp. (AOA 8%)§	6,801 (5,791–7,712)
*Shigella* spp. (AOA 40%)§	626 (487–797)
STEC (AOA 21%)‡§	3-y data, South Australia only, 37 (18–51)
*Yersinia* spp. (AOA 2%)§	157 (74–212)

*STEC, Shiga toxin–producing *Escherichia coli*; AOA, adjusted for overseas acquired; NSW, New South Wales. †Proportion applied to estimate of total gastroenteritis in Australia. ‡Population factor applied. §Underreporting factors for outbreaks and moderate illness applied.

**Table A2:** Underreporting factors for moderate, bloody, and serious illness

Severity of illness	Information used for estimation	Estimate (credible interval)
Moderate illness	Australian surveillance data, Melbourne Water Quality Survey (17), Victorian *Salmonella* outbreak data, National Gastroenteritis Survey, Hunter *Salmonella* case-control study (24)	1 in 15 (5–25)
Bloody diarrhea	National Gastroenteritis Survey	1 in 9 (1–17)
Serious illness	Mead et al., 1999 (6)	1 in 2 (1–3)
